# Is Fixation of the Ulnar Styloid Necessary After Distal Radius Fracture Stabilization?

**DOI:** 10.7759/cureus.107274

**Published:** 2026-04-18

**Authors:** Bhavya Sirohi, Mohd A Jafri, Kumud Kishlaya, Swapnil B Bhalerao, Parth Hemal Bhagat, Shagun Chandramohan Yadav, Vijay Raisahab Yadav

**Affiliations:** 1 Orthopaedics, Military Hospital, Agra, IND; 2 Orthopaedics, Command Hospital, Udhampur, IND; 3 Orthopaedics, Military Hospital, Jammu, IND; 4 Orthopaedics, Military Hospital, Jodhpur, IND; 5 Orthopaedics and Traumatology, SMBT Hospital and Institute of Medical Sciences and Research Centre (IMSRC), Nashik, IND; 6 Orthopaedics, SMBT Hospital and Institute of Medical Sciences and Research Centre (IMSRC), Nashik, IND

**Keywords:** fernandez type i distal radius fracture, fixation, fracture distal radius, outcome, ulnar styloid fracture

## Abstract

Introduction

Fractures of the distal radius are frequently accompanied by fractures of the ulnar styloid process. Because the triangular fibrocartilage complex attaches to the base of the ulnar styloid and contributes to the stability of the distal radioulnar joint (DRUJ), the optimal management of these fractures remains controversial. While some surgeons advocate fixation of the ulnar styloid fragment to restore joint stability, others believe that fixation may not significantly influence clinical outcomes.

Methods

This retrospective comparative study included patients presenting with Fernandez type I distal radius fractures associated with a base fracture of the ulnar styloid. Patients were divided into two groups based on the treatment received: those who underwent fixation of the ulnar styloid fragment and those managed without fixation. All distal radius fractures had been stabilized using percutaneous crossed Kirschner wires. In the fixation group, the ulnar styloid fragment had been additionally stabilized using a tension-band wiring technique. Clinical and follow-up data were retrieved from medical records, with a minimum follow-up duration of 12 months. Outcomes were assessed using the Visual Analogue Scale (VAS) for pain, the Quick Disabilities of the Arm, Shoulder, and Hand (Quick-DASH) questionnaire, the Mayo Wrist Performance Score, wrist range of motion, and grip strength.

Results

A total of 121 patients were included (fixation: 63; without fixation: 58). Quick-DASH scores were slightly higher in the fixation group at three months (34.4 ± 13.5 vs. 32.9 ± 5.7, p = 0.027) and six months (29.8 ± 18.2 vs. 19.3 ± 8.2, p = 0.001), but no difference was observed at 12 months (12.7 ± 7.7 vs. 6.4 ± 2.1, p = 0.198). Mayo Wrist Scores, VAS pain scores, wrist range of motion, and grip strength were comparable between the groups at all time points (all p > 0.05).

Conclusion

Fixation of the ulnar styloid fragment did not provide a measurable long-term functional advantage after distal radius fracture stabilization. Routine surgical fixation of the ulnar styloid may therefore be unnecessary in patients with a stable DRUJ.

## Introduction

Distal radius fractures are among the most common injuries affecting the upper extremity and represent a substantial proportion of cases presenting to emergency departments, occurring in approximately one out of every six patients with fractures [1]. These injuries are frequently accompanied by fractures of the ulnar styloid process, which have been reported in approximately 50%-65% of patients with distal radius fractures [2,3].

From an anatomical and biomechanical perspective, the ulnar styloid plays an important role in the stability of the distal radioulnar joint (DRUJ) because the triangular fibrocartilage complex (TFCC) attaches to its base and contributes to maintaining joint stability [4]. Injury at this site may therefore theoretically influence wrist function and DRUJ stability following distal radius fractures.

Management strategies for ulnar styloid fractures vary widely and may include conservative treatment or surgical fixation using different techniques such as plates, external fixation, or pin-and-wire constructs [5]. Several studies have reported that fixation of the ulnar styloid fracture accompanying distal radius fractures does not significantly influence the final functional outcome [3,5,6]. In contrast, other investigations have suggested that fractures involving the base of the ulnar styloid may increase the likelihood of DRUJ instability due to disruption of the TFCC attachment [7].

Because of these conflicting findings, the necessity of routine fixation of the ulnar styloid fracture remains uncertain. Therefore, the present retrospective study was conducted to compare the clinical and functional outcomes of patients with distal radius fractures treated either with fixation or without fixation of the associated ulnar styloid fracture.

## Materials and methods

Study design and participants

This was a retrospective, single-center comparative study conducted to evaluate the outcomes of ulnar styloid fixation in patients with distal radius fractures. Medical records of patients treated for distal radius fractures associated with a base fracture of the ulnar styloid were reviewed. All outcome assessments were performed by independent evaluators who were blinded to the treatment allocation of each group, in order to reduce assessment bias.

The inclusion criteria consisted of adult patients with Fernandez type I distal radius fractures, according to the Fernandez classification [8], accompanied by a base fracture of the ulnar styloid and a stable DRUJ. In our study, DRUJ stability was assessed clinically at presentation using standard bedside methods, primarily the ballottement (DRUJ stress) test, with comparison to the contralateral side. Cases with any clinical suspicion of instability were excluded. TFCC injury was assessed with MRI, and large tears (type 1B and above) were excluded from the study. The exclusion criteria included patients younger than 18 years, the presence of open fractures, and a history of previous wrist or hand surgery or deformity.

**Figure 1:**
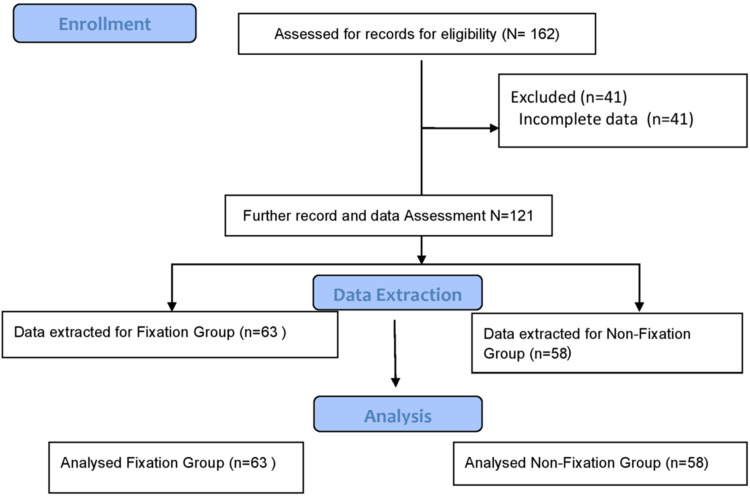
Flow diagram of patient selection

Patients were categorized into two groups based on the treatment received: those who underwent fixation of the ulnar styloid fracture and those in whom the ulnar styloid fracture was not fixed. A total of 162 patients with distal radius fractures were identified from institutional records and screened for eligibility. Of these, 23 patients were excluded due to not meeting the inclusion criteria (n = 16), incomplete records (n = 3), or the presence of a second fracture (n = 4). The remaining 139 patients were included in the retrospective analysis. Based on the treatment received, 72 patients were categorized into the fixation group and 67 into the non-fixation group. Follow-up data were incomplete for nine patients in the fixation group and four patients in the non-fixation group, leaving 63 and 58 patients, respectively, for final analysis (Figure 1).

Power analysis

Based on previously reported data by Moradi et al. [5], assuming a standard deviation of 13 and a minimum clinically important difference of 10 points in the Quick Disabilities of the Arm, Shoulder, and Hand (Quick-DASH) score, the estimated sample size required to achieve 80% power at a two-sided significance level of 0.05 was approximately 26 patients per group. However, no formal a priori sample size calculation was performed, and the study sample was determined by available cases.

Surgical intervention

All distal radius fractures were stabilized using percutaneous crossed Kirschner wire fixation. In patients in whom fixation of the ulnar styloid was performed, the fragment was additionally stabilized through a small longitudinal incision over the ulnar styloid using a tension-band wiring technique with Kirschner wires. Following surgical stabilization, both groups were immobilized in a long-arm cast with the forearm maintained in supination for six weeks.

Postoperative care and follow-up

Postoperative management was similar for both groups. During the immobilization period, patients were instructed to perform active exercises of the fingers and shoulder to prevent stiffness. After cast removal, gradual wrist mobilization was initiated.

Patients were followed up at four, eight, 12, 24, and 48 weeks after surgery. Clinical data were obtained from follow-up records. Pain intensity was assessed using the Visual Analogue Scale (VAS). Functional outcomes were evaluated using the Quick-DASH questionnaire and the Mayo Wrist Performance Score. Wrist range of motion and grip strength were also assessed and compared with the contralateral side. Outcome measures were recorded at three, six, and 12 months.

Outcome measures

Pain severity was assessed using the 10-point VAS, where 0 indicates no pain and 10 represents the worst pain experienced by the patient [9]. VAS scores were obtained from documented clinical records at predefined follow-up intervals.

Upper extremity functional disability was evaluated using the Quick-DASH questionnaire, an 11-item patient-reported outcome measure designed to assess functional limitations of the upper limb. Each item has five response options, and the final score ranges from 0 (no disability) to 100 (maximum disability) [10]. Recorded Quick-DASH scores were retrieved from follow-up documentation.

Wrist function was assessed using the Mayo Wrist Performance Score, which evaluates pain, range of motion, grip strength, and return to work. The total score ranges from 0 to 100, with higher scores representing better functional outcome [11]. These scores were obtained from clinical assessment records.

Grip strength was measured using a hydraulic grip dynamometer (Medilab India, Bengaluru, India). Each measurement had been performed at least three times, and the final value was expressed as the ratio of the affected hand strength to that of the unaffected hand, as documented in patient records.

Wrist range of motion, including flexion-extension and radial-ulnar deviation, had been measured in degrees using an orthopedic goniometer for both the affected and contralateral wrists. These measurements were extracted from follow-up records.

Statistical analysis

Statistical analysis was performed using IBM SPSS Statistics version 16.0 (SPSS Inc., Chicago, IL, USA). Categorical variables, including sex, affected side, and cause of injury, were analyzed using Fisher’s exact test. Continuous and ordinal variables were compared between groups using Student’s t-test or the Mann-Whitney U test, depending on the distribution of the data. A p-value < 0.05 was considered statistically significant.

Sample size consideration

As this was a retrospective study, no a priori sample size calculation was performed. However, a post hoc assessment of sample adequacy was conducted. The sample size required to detect a significant difference in functional outcomes between patients treated with ulnar styloid fixation and those treated without fixation can be estimated using the formula for comparison of two independent means:

$n=\frac{2\sigma^{2}\left( Z_{\alpha/2}+Z_{\beta} \right)^{2}}{d^{2}}$

where σ represents the expected standard deviation of the outcome measure and d represents the minimum clinically significant difference between the two groups. Based on previously published data evaluating functional outcomes following distal radius fractures with associated ulnar styloid fractures, assuming a standard deviation of 13 and a minimum clinically important difference of 10 points in the Quick-DASH score, the calculated minimum sample size was approximately 26 patients per group, as reported by Moradi et al. [5]. The present study included 63 patients in the fixation group and 58 in the non-fixation group, which exceeds the estimated minimum sample size, indicating adequate statistical power to detect clinically meaningful differences.

## Results

A total of 121 patients were included in the study, with 63 undergoing ulnar styloid fixation and 58 managed without fixation. The groups were comparable with respect to baseline characteristics.

Baseline demographic characteristics were comparable between the fixation (n = 63) and non-fixation (n = 58) groups. There were no significant differences between groups with respect to age, sex distribution, cause of injury, or affected side (all p > 0.05).

A total of 121 patients were included in the study, comprising 69 men (57.0%) and 52 women (43.0%). The most common cause of injury was falling (81.0%), followed by motor vehicle accidents (19.0%). The left wrist was more frequently affected (62.8%) than the right (37.2%), and the mean age was 52.2 ± 15.4 years (Table 1).

**Table 1:** Demographic characteristics SD: standard deviation

Variable	Category	Value
Sex (N, %)	Male	69 (57.0)
	Female	52 (43.0)
Cause of injury (N, %)	Motor vehicle accident	23 (19.0)
	Fall	98 (81.0)
Affected side (N, %)	Right	45 (37.2)
	Left	76 (62.8)
Age (mean ± SD)		52.2 ± 15.4

The Mayo Wrist Score was comparable between the fixation and non-fixation groups (58.3 ± 10.9 vs. 59.8 ± 5.6, p = 0.504). Early postoperative DASH scores at three months were slightly higher in the fixation group (34.4 ± 13.5 vs. 32.9 ± 5.7), but this difference was not statistically significant (p = 0.427). However, at six months, the difference became statistically significant, with higher scores in the fixation group (29.8 ± 18.2 vs. 19.3 ± 8.2, p = 0.001). However, at the end of 12 months, DASH scores continued to improve in both groups, with no significant difference among groups (12.7 ± 7.7 vs. 6.4 ± 2.1, p = 0.134).

Preoperative VAS scores were comparable between the two groups (3.03 ± 1.57 vs. 3.55 ± 1.46, p = 0.066). At six months, VAS scores remained similar without a statistically significant difference (4.44 ± 2.21 vs. 4.12 ± 0.28, p = 0.265). At 12 months, however, VAS scores were significantly higher in the fixation group (4.07 ± 1.66 vs. 2.50 ± 0.96, p < 0.001) (Table 2).

**Table 2:** Comparison of demographic characteristics and clinical outcomes between the fixation and non-fixation groups Continuous variables are expressed as mean ± standard deviation (SD) and compared using the independent samples t-test. Categorical variables are expressed as frequency (percentage) and compared using the Chi-squared test or Fisher’s exact test *Fisher’s exact test was used where expected cell counts <5; a two-tailed p-value < 0.05 was considered statistically significant; normality was assessed using the Shapiro-Wilk test VAS: Visual Analogue Scale; DASH: Disabilities of the Arm, Shoulder, and Hand

Variable	Fixation (n = 63)	Without fixation (n = 58)	Test statistic	p-value
Age (years)	51.5 ± 14.8	52.9 ± 16.2	t = -0.50	0.754
Cause of injury				0.868*
Road accident	11 (17.5%)	15 (25.9%)	χ² = 0.03	
Fall	52 (82.5%)	43 (74.1%)	-	
Affected side				0.830*
Right	43 (68.3%)	41 (70.7%)	χ² = 0.05	
Left	20 (31.7%)	17 (29.3%)	-	
Gender				0.089*
Female	25 (39.7%)	27 (46.6%)	χ² = 2.88	
Male	38 (60.3%)	31 (53.4%)		
Mayo Wrist Score	58.3 ± 10.9	59.8 ± 5.6	t = -0.94	0.504
DASH score (3 months)	34.4 ± 13.5	32.9 ± 5.72	t = 0.80	0.427
DASH score (6 months)	29.8 ± 18.2	19.3 ± 8.23	t = 3.92	0.001
DASH score (12 months)	12.7 ± 7.66	6.42 ± 2.13	t = 5.98	0.134
VAS (before surgery)	3.03 ± 1.57	3.55 ± 1.46	t = -1.86	0.066
VAS (6 months)	4.44 ± 2.21	4.12 ± 0.28	t = 1.12	0.265
VAS (12 months)	4.07 ± 1.66	2.50 ± 0.96	t = 6.16	<0.001
Wrist flexion (°)	43.7 ± 7.88	42.8 ± 8.03	t = 0.62	0.536
Wrist extension (°)	35.7 ± 12.3	37.3 ± 11.7	t = -0.73	0.467
Forearm supination (°)	44.5 ± 19.6	47.3 ± 14.7	t = -0.90	0.371
Forearm pronation (°)	76.6 ± 10.3	74.4 ± 12.7	t = 1.06	0.292
Grip strength	42.3 ± 21.4	40.3 ± 13.2	t = 0.60	0.552
Wrist ulnar deviation (°)	16.9 ± 5.38	16.6 ± 6.24	t = 0.28	0.780
Wrist radial deviation (°)	11.7 ± 3.63	11.8 ± 3.34	t = -0.15	0.880

Wrist range of motion, including flexion, extension, supination, pronation, radial deviation, and ulnar deviation, showed no statistically significant differences between the two groups (all p > 0.05). Grip strength was also comparable between groups (42.3 ± 21.4 vs. 40.3 ± 13.2, p = 0.552) (Table 2).

These findings suggest that ulnar styloid fixation did not confer a significant long-term advantage in wrist function, pain reduction, or grip strength. Although differences in early Quick-DASH scores were observed, these did not persist at the final follow-up (Table 2).

## Discussion

The present retrospective study evaluated the impact of ulnar styloid fixation on clinical and functional outcomes following distal radius fracture stabilization. The findings indicate that fixation of the ulnar styloid fragment does not provide a significant long-term benefit. Although slightly worse early Quick-DASH scores were observed in the fixation group, these differences were not sustained at final follow-up, and overall outcomes remained comparable between the groups.

Pain outcomes, as assessed by the VAS, were similar in both groups throughout follow-up. While marginally higher pain scores were noted in the fixation group, these differences did not reach statistical significance. This observation may be related to additional soft tissue handling and surgical dissection required for fixation of the ulnar styloid fragment. Previous studies have suggested that postoperative pain is more closely associated with the adequacy of distal radius fracture stabilization rather than the management of the ulnar styloid fracture (Zyluk et al. [6]). Furthermore, concomitant injury to the TFCC, particularly in high-energy trauma, may contribute to persistent ulnar-sided wrist pain irrespective of fixation strategy (Sachar [12]). Similar findings have been reported by Zenke et al. [2], who observed no significant difference in pain outcomes between the fixation and non-fixation groups.

Functional recovery, evaluated using the Quick-DASH score, demonstrated improvement over time in both groups. Although early postoperative scores were higher in the fixation group, no significant difference was observed at one year. These findings are consistent with previous reports indicating that fixation of the ulnar styloid does not substantially influence upper extremity function (Zenke et al. [2]).

In the present study, wrist range of motion and grip strength were comparable between the fixation and non-fixation groups. These findings are consistent with previous studies demonstrating that the presence or management of an ulnar styloid fracture does not significantly affect wrist mobility or strength following distal radius fracture treatment, as reported by Buijze and Ring [13].

Variability in reported outcomes across studies may be attributed to differences in fracture configuration, surgical techniques, and patient characteristics. It has been suggested that fractures involving the base of the ulnar styloid may be associated with a higher risk of DRUJ instability due to disruption of the TFCC insertion. In such cases, selective fixation may be considered to restore joint stability and potentially improve outcomes, as highlighted by Sawada et al. [14].

Supporting this, Wijffels and Ring [15] reported that the presence, type, or union status of ulnar styloid fractures had no measurable impact on functional outcomes. Similarly, Kim et al. [4] found that non-union of the ulnar styloid did not adversely affect wrist motion, strength, or pain. A meta-analysis by Mulders et al. [16] also demonstrated that while a small difference in DASH scores may favor patients without ulnar styloid fractures, other parameters such as pain, grip strength, wrist motion, and DRUJ stability remain comparable. Notably, most of these studies did not specifically evaluate the effect of surgical fixation of the ulnar styloid fragment.

Similarly, Velmurugesan et al. [17] reported that ulnar styloid base fractures are associated with TFCC injury and DRUJ instability. They found no significant difference in pain or functional outcomes between surgical and conservative management following distal radius fixation. However, conservative treatment had higher non-union rates, and non-union was associated with worse pain and functional outcomes, with pre-operative displacement predicting non-union. Afifi and Mansour [18], in a randomized controlled trial, found no significant difference in DASH scores, wrist function, pain, grip strength, or range of motion between fixation and non-fixation of ulnar styloid base fractures. Both groups demonstrated comparable outcomes at final follow-up. They concluded that fixation is not necessary when the DRUJ is stable following distal radius fixation.

Overall, the findings of this study suggest that routine fixation of the ulnar styloid is not necessary in the setting of a stable DRUJ. The absence of meaningful differences in long-term outcomes, combined with the potential for additional surgical morbidity, supports a more selective approach to management. Given the retrospective nature of the study, causal relationships cannot be definitively established, and the results should be interpreted with consideration of potential selection bias and confounding factors.

Limitations

This study has several limitations. Its retrospective, non-randomized design introduces potential selection bias and unmeasured confounding, particularly as the decision to fix or not fix the ulnar styloid was not randomized. Being a single-center study, the findings may not be generalizable, and the use of long-arm cast immobilization further limits applicability to centers employing volar plating with early mobilization.

Only pinning, casting, and tension-band wiring were used, while more rigid fixation methods (e.g., screws or plates) were not evaluated and may yield different outcomes. Importantly, DRUJ stability was not assessed at final follow-up despite being the central clinical question, and the union status of the ulnar styloid was not reported in either group. The lack of inter-rater reliability assessment for range-of-motion measurements introduces potential measurement bias.

Statistically, post hoc power analysis based on observed effect sizes may overestimate true power, and no adjustment for multiple comparisons was performed despite multiple outcomes and time points, increasing the risk of type I error. Although normality assumptions were tested, wide variability in some measures suggests possible deviations, warranting cautious interpretation. Finally, inclusion of only Fernandez type I distal radius fractures and a follow-up limited to one year restrict broader applicability; longer follow-up and more comprehensive assessment (e.g., arthroscopy) would strengthen conclusions.

## Conclusions

In conclusion, this retrospective single-center study suggests that, in patients with a stable DRUJ, fixation of the ulnar styloid does not result in superior wrist pain, range of motion, or functional outcomes following distal radius fracture stabilization. The fixation group demonstrated statistically worse DASH scores at six and 12 months; however, these differences were small and did not exceed the minimal clinically important difference. By the final follow-up, no meaningful difference was observed between groups. These findings suggest that routine fixation of ulnar styloid fractures may not be necessary in all patients with a stable DRUJ. Further prospective studies with larger sample sizes, longer follow-up, and assessment of radiographic and soft-tissue factors are needed.
